# Supplementary material for: The role of race and scientific trust on support for COVID-19 social distancing measures in the United States
Source: PLoS One. 2021 Jul 9;16(7):e0254127. doi: 10.1371/journal.pone.0254127 (PMC8270185; doi:10.1371/journal.pone.0254127)
Supplement: S1 Appendix — (PDF) [file pone.0254127.s001.pdf]

Appendix S1

Variable coding schemes and question wordings

Scientific Trust Variable Construction Questions: Taken from Pew `American National Trends Panel Survey` Wave 42 filed from January 7–21, 2019), we use the following questions to estimate latent scientific trust of respondents. Note that these questions are asked over a year prior to the onset of the COVID-19 pandemic in the United States, thus alleviating endogeneity concerns between pandemic conditions and attainment of scientific knowledge. Below we show the individual questions used to create our latent measure of scientific knowledge, with the parenthesis indicating the percentage of respondents chose the response as an answer to the respective question. Note that we code all the questions to reflect higher degrees of adherence to scientific trust prior to the construction of our latent scientific trust variable in a two-dimensional confirmatory factor analysis.[1]

1. How much confidence, if any, do you have in each of the following to act in the best interest of the public (medical scientists)?
   - A great deal of confidence (36%)
   - A fair amount of confidence (52%)
   - Not too much confidence (10%)
   - No confidence at all (1%)
   - Refused (0%)
2. How much confidence, if any, do you have in each of the following to act in the best interest of the public (scientists)?[2]
   - A great deal of confidence (36%)
   - A fair amount of confidence (51%)
   - Not too much confidence (10%)
   - No confidence at all (2%)
   - Refused (0%)
3. Which of the following statements comes closer to your own view, even if neither is exactly right?
   - Scientists should take an active role in public policy debates about scientific issues (61%)

1. For coding of the questions used to derive the dependent variables used in this study, please see the comprehensive discussion in the research design section of the manuscript.

2. As mentioned in the manuscript, the first two questions were split in the sample, with half the sample being asked to rate "medical scientists" while the second half was asked to rate "scientists." We combine both of these measures to measure overall trust in "scientists" to act in the best interests of the public as stated by the survey question. Given the identical responses in the survey questions, this approach is methodologically sound.

- Scientists should focus on establishing sound scientific facts and stay out of public policy debates (38%)
- Refused (1%)

4. In general, would you say scientific experts are...

   - Usually BETTER at making good policy decisions about scientific issues than other people (45%)
   - Usually WORSE at making good policy decisions about scientific issues than other people (6%)
   - NEITHER BETTER NOR WORSE at making good policy decisions about scientific issues than other people (48%)
   - Refused (1%)

5. Which of the following best describes what you think about the scientific method?

   - The scientific method generally produces accurate conclusions (64%)
   - The scientific method can be used to produce any conclusion the research wants (35%)
   - Refused (1%)

6. Which of these statements comes closer to your own view, even if neither is exactly right?

   - Scientists make judgments based solely on the facts (55%)
   - Scientists' judgments are just as likely to be biased as other people's (44%)
   - Refused (1%)

7. How important do you think each of the following types of scientific research is for society?

   - Essential (67%)
   - Important, but not essential (27%)
   - Not too important (4%)
   - Not important at all (1%)
   - Refused (1%)

8. Scientific research that advances knowledge, even if there are no immediate benefits?

   - Essential (50%)
   - Important, but not essential (42%)
   - Not too important (7%)
   - Not important at all (1%)
   - Refused (1%)

Elected Officials Trust: How much confidence, if any, do you have in each of the following to act in the best interests of the public (Elected officials)? Responses to this question were coded 4 (a great deal of confidence, 3%), 3 (a fair amount of confidence, 31%), 2 (not too much confidence, 51%), and 1 (no confidence at all, 14%).

Media Institutional Trust: How much confidence, if any, do you have in each of the following to act in the best interests of the public (the news media)? Responses to this question were coded 4 (a great deal of confidence, 9%), 3 (a fair amount of confidence, 39%), 2 (not too much confidence, 32%), and 1 (no confidence at all, 19%).

Race: Series of dummy variables were used to code a respondent's race, with the reference category being if a respondent's identified as "white non-Hispanic." Thus dummy variables were coded if respondents identified as "black non-Hispanic" (coded as African-American respondents), "Hispanic" (coded as Hispanic-American respondents), and "Asian or Asian-American" (coded as Asian-American respondents). Note that the coding for Hispanic-Americans were recovered from an additional variable measuring respondent ethnicity per standard demographic convention.

Gender: Pew binary measure coded 1 for women and 0 for men.

Self-Reported Partisanship: Nominal variable coded 1 for Republican, 2 for Independent, and 3 for Democrat.

Ideology: Self-identified symbolic ideology coded on a five-point ordinal scale from 1 (very conservative) to 5 (very liberal). A value of 3 indicates a self-identified moderate.

Age: Self-reported age variable coded on a 4-point ordinal scale in the following fashion: 1 (18-29), 2 (30-49), 3 (50-64) and 4 (65+).

Education: Self-reported highest educational attainment coded on a standard six-point ordinal scale in the following fashion: 1 (less than high school), 2 (high school graduate), 3 (some college), 4 (associate's degree), 5 (college degree), and 6 (post-graduate degree).

Income: Self-reported income on a nine-point ordinal scale recorded in the following fashion: 1 (less than $ 10,000), 2 ($10,000-$20,000), 3 ($20,000-$30,000), 4 ($30,000-$40,000), 5 ($40,000-$50,000), 6 ($50,000-$75,000), 7 ($75,000-$100,000), 8 ($100,000-$150,000), and 9 ($150,000 or more).

Census Region: Contextual variable coded in a series of three dummy variables indicating residence in the Midwest, South, and West (omitted baseline category is the Northeast region).
