# Supplementary figures and images for: The role of race and scientific trust on support for COVID-19 social distancing measures in the United States
Source: PLoS One. 2021 Jul 9;16(7):e0254127. doi: 10.1371/journal.pone.0254127 (PMC8270185; doi:10.1371/journal.pone.0254127)

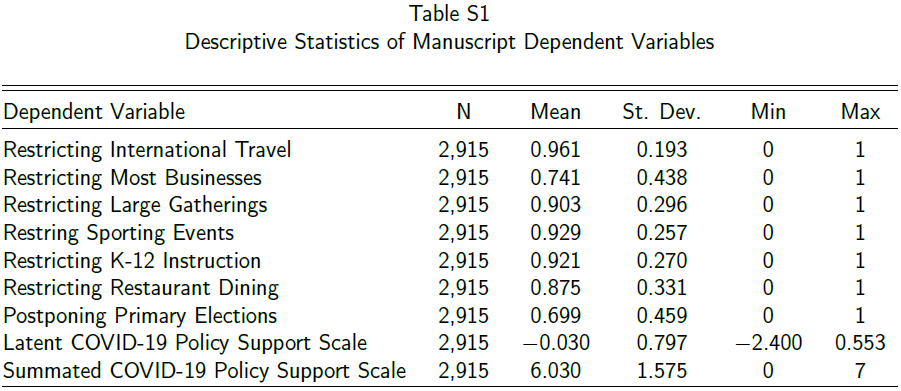

Supplement: S1 Table — (PNG) [file pone.0254127.s002.png]

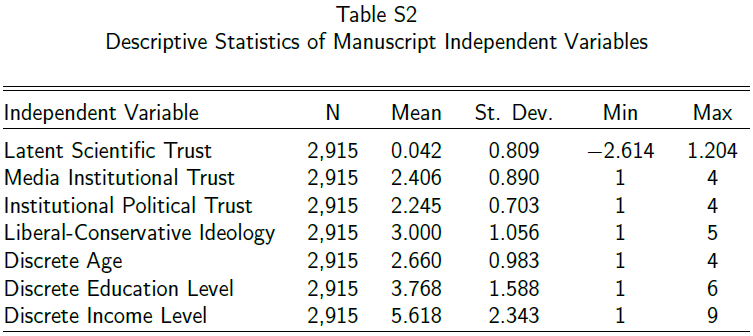

Supplement: S2 Table — (PNG) [file pone.0254127.s003.png]

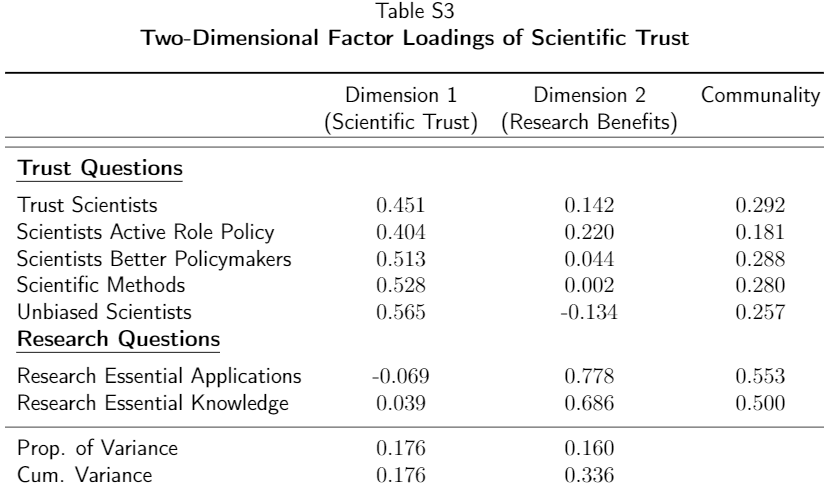

Supplement: S3 Table — (PNG) [file pone.0254127.s004.png]

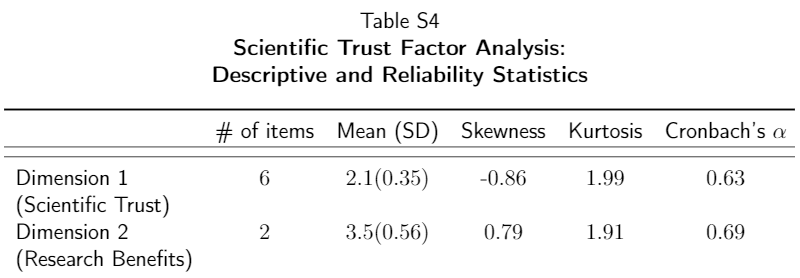

Supplement: S4 Table — (PNG) [file pone.0254127.s005.png]

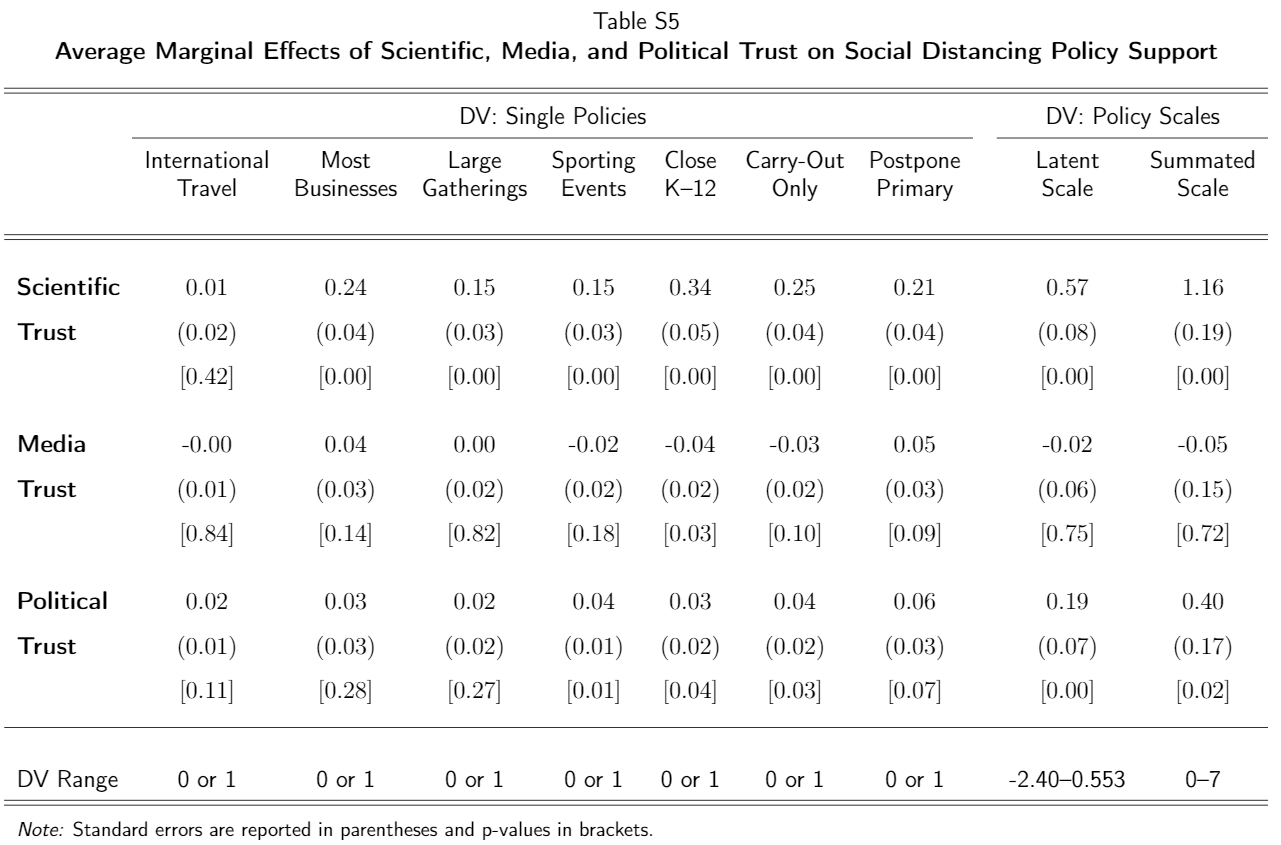

Supplement: S5 Table — (PNG) [file pone.0254127.s006.png]

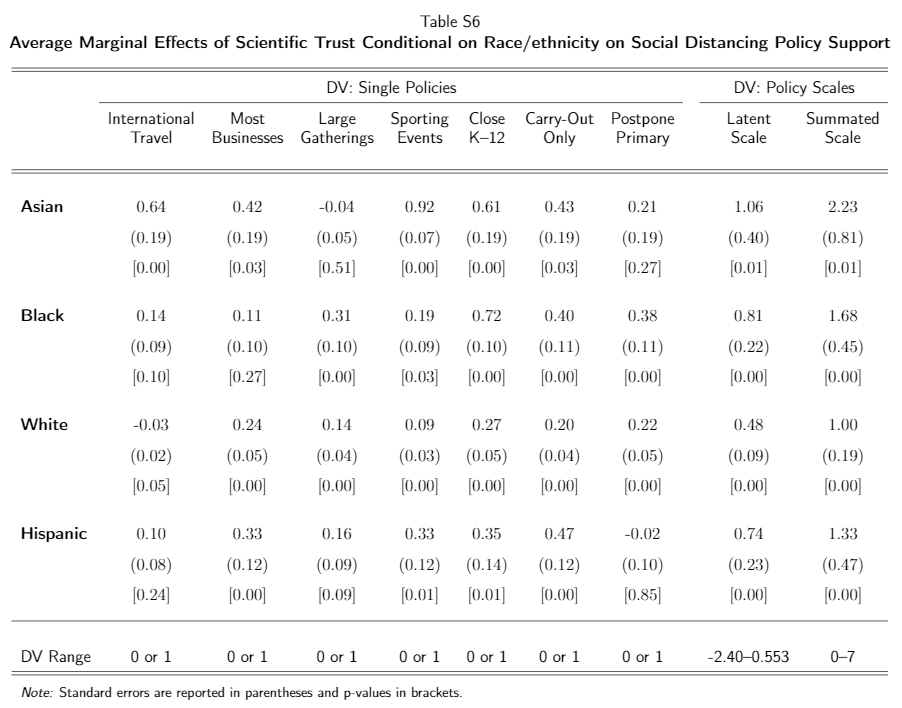

Supplement: S6 Table — (PNG) [file pone.0254127.s007.png]

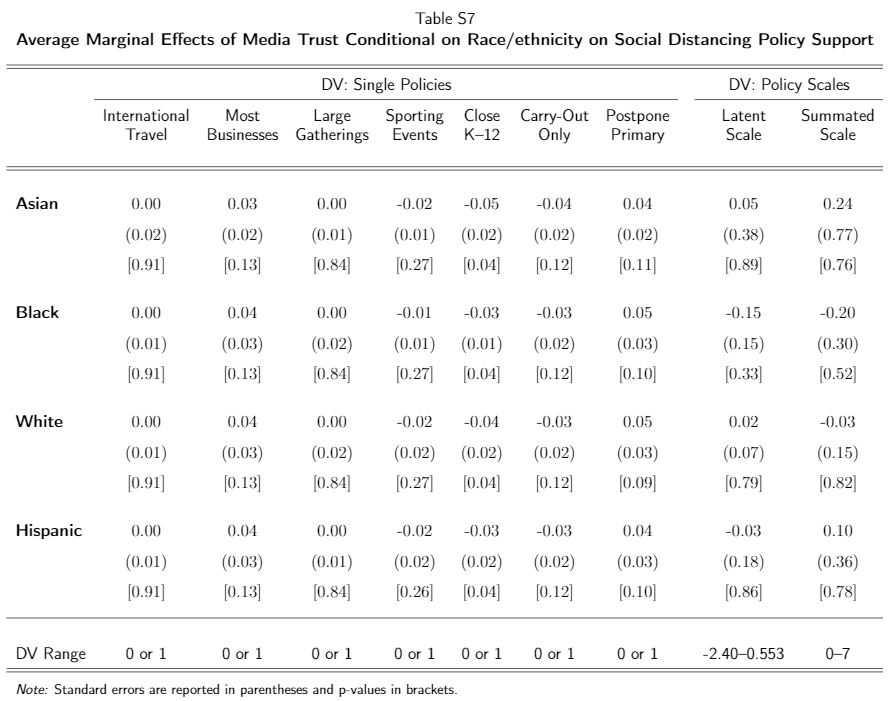

Supplement: S7 Table — (PNG) [file pone.0254127.s008.png]

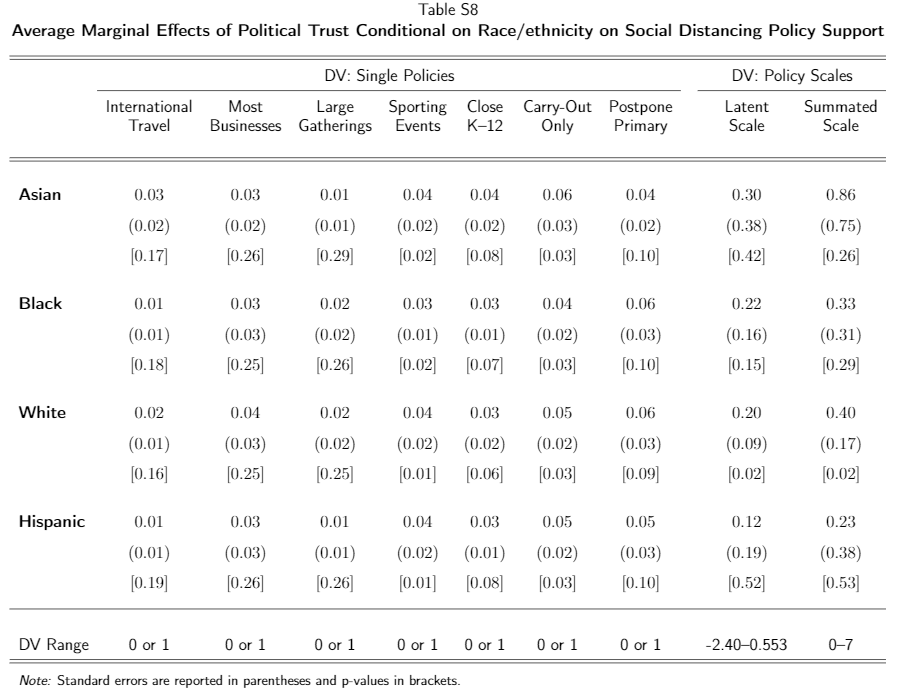

Supplement: S8 Table — (PNG) [file pone.0254127.s009.png]

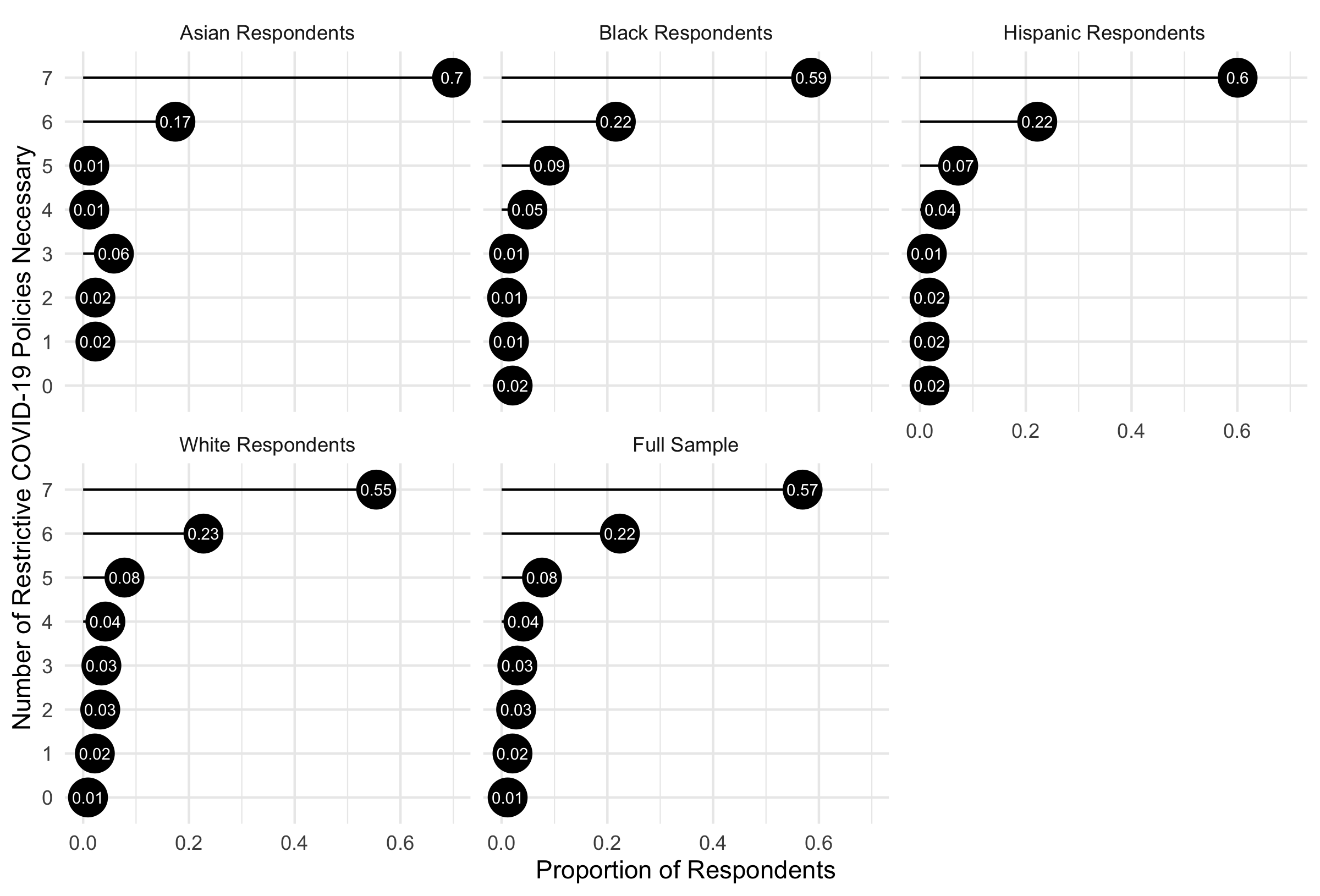

Supplement: S1 Fig — (PNG) [file pone.0254127.s010.png]

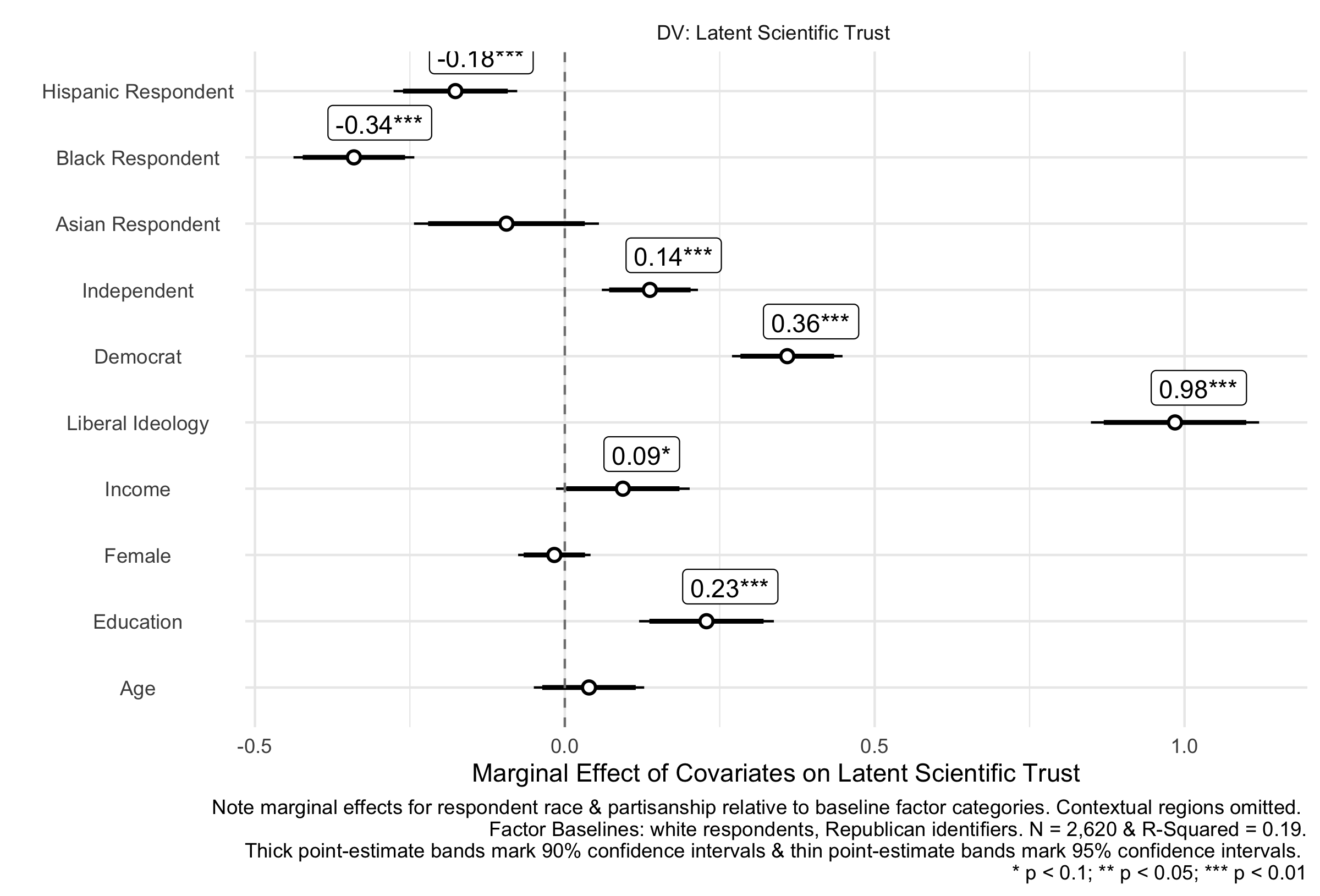

Supplement: S2 Fig — (PNG) [file pone.0254127.s011.png]
