# Supplementary material for: The role of race and scientific trust on support for COVID-19 social distancing measures in the United States
Source: PLoS One. 2021 Jul 9;16(7):e0254127. doi: 10.1371/journal.pone.0254127 (PMC8270185; doi:10.1371/journal.pone.0254127)
Supplement: Attachment — Submitted filename: Response to Reviewers.pdf [file pone.0254127.s012.pdf]

PONE-D-21-06826

The role of race and scientifict trust on support for COVID-19 social distancing measures in the United States

Revise & Resubmit Manuscript Memo

We would like to thank the reviewers and the editor for their careful reading of our manuscript and for their insightful comments. Their suggestions motivated a substantial revision of the manuscript which, in our view, greatly strengthens both its clarity and contribution. The major changes in this new version of the manuscript are as follows:

1 Major Changes

- One of the more substantive changes of the revised manuscript relates to the point brought up by the editor with respect to framing the manuscript's empirical contribution into the broader context of how governments seek to contain the broader COVID-19 pandemic. We agree with the editor's comment that we must stress the importance of our focus on support for public policies aimed at mitigating the COVID-19 pandemic. To that end, we strengthen the motivation of our manuscript by stressing the classic representational framework between citizens and government, that citizens delegate to governments to solve societal collective action problems such as the COVID-19 pandemic. Given that governments are comprised of legislators, it is critical that policies garner sufficient support to create an electoral incentive for office-seeking politicians to implement policies that are popular with the mass public. In our manuscript, we identify that within the context of the COVID-19 pandemic, scientific trust provides a mechanism by which support for critical policies aimed at ensuring social distancing rises, particularly within minority communities that are disproportionately affected by the pandemic. In turn, this trust serves as a basis for increasing support for public health policies during a pandemic which provides an incentive for the implementation of these policies by politicians. Indeed, the literature identifies that implementation of these social distancing policies are critical towards mitigating variation in compliance of social distancing behavior in the mass public and, ultimately, halting the transmission of the SARS-CoV-2 virus causing deadly COVID-19 pandemic. Taken together, we strengthen our manuscript by placing our study within the broader literature on representation and how policy support in the mass public serves as a critical incentive motivating governments to act, particularly during a pandemic which requires pronounced and swift governmental action within the realm of public health.

- A second major change in the revised manuscript is clarification of language regarding our research design as articulated by the reviewers. Specifically, we add language that walks the reader through the specification of our aggregate models predicting the severity of the COVID-19 pandemic across minority counties and our individual-level models predicting policy support for critical social distancing public health policies in the mass public. We also emphasize the descriptive nature of our analysis and add additional information regarding the manuscript empirics with additional tables and latent variable measurement descriptions in the manuscript supporting information.

- We also add relevant policy implications of our study while also noting future work given limitations of this study along the lines suggested by the reviewers.
- We add additional language to clarify the link between the county-level models and the individual-level study. Building off the previous change, we add additional context linking how scientific trust may help address the standing racial disparities described in the motivating models of the manuscript. We also add relevant literature to this end and along the lines suggested by the reviewers.

We would like to thank the reviewers again for their insightful suggestions as these major changes help strengthen the manuscript and highlight its contribution to our scholarly understanding of the racial differences in how scientific trust affects social distancing measures in the United States. In the subsequent sections, we provide a more detailed response to each of the reviewers' and editor's comments. In each case, we present the comments in italics, followed by our response.

2 Reviewer 1

> *I liked this paper and felt it was useful, well written, and well executed. I nevertheless have some areas for improvement to suggest:*

2.1 General Framing

> *The authors need to make the historical and current context for ethnic minorities come earlier in the paper and be sprinkled more throughout. Otherwise this comes across as a bit deficit framed, i.e. lack of trust rather than situating the trust in historical racism and current racism.*
>
> *Sometimes this also comes across as a little naïve e.g. p 3 line 87, when the authors start on the racial disparities -there is a huge body of work on racism in science, govt, medicine that needs to be drawn on.*
>
> *Sometimes the authors also refer to historic racism, when again, it is clear racism is still an issue. This should be framed as historic and current.*

We concur with the reviewer that there are more modern factors that contribute to medical and scientific trust gap among White, Black and Hispanic Americans. As such, we have added a discussion on more present-day factors that impact distrust, including exposure to news media as well as persona experiences with medical professionals. For example, we cite the important work by Hammond (2010) [1] as well as Williams (2019)[2] and Williams (2021)[3] to emphasize how

[1] Hammond, W. P. (2010). Psychosocial correlates of medical mistrust among African American men. American journal of community psychology, 45(1-2), 87-106.

[2] Williamson, L. D., Smith, M. A., & Bigman, C. A. (2019). Does discrimination breed mistrust? Examining the role of mediated and non-mediated discrimination experiences in medical mistrust. Journal of health communication, 24(10), 791-799.

[3] Williamson, L. D. (2021). Beyond Personal Experiences: Examining Mediated Vicarious Experiences as an Antecedent of Medical Mistrust. Health Communication, 1-14.

racism and personal experiences with medical doctors account for the racial trust gap between White Americans and minorities.

2.2 Empirics & Presentation

> *Table 1–these by county analyses could be more firmly linked in - why are they needed? don't we know already there are these disparities? Could be woven in a bit more and more mention made of the need for these analyses.*

We thank the reviewer for pointing us in this direction, we agree that linking this analysis in more is warranted. Please see the track changes for specifics, but we have added a paragraph that better explains the value that this analysis brings to both this project specifically but also research on racial disparities related to COVID-19 generally.

> *How was race measured? What about mixed race people, what do they count as? At times this work read a bit USA-centric (also, make it clear what K-12 is please). Similarly, I was wondering why the authors use the term "citizens" - do they mean literal citizens? Residents? Why not just people?*

We thank the reviewer for this important point and inquiry. First, race is measured according to standard survey practices in that racial identification is self-reported and we make a note of this in the revised version of the manuscript. Indeed, Pew measures race in the standard way by using two variables to capture racial identification and Hispanic origin which is consistent with the method used by the Census. As such, we combine both measures to identify distinct racial identification across White, African-American, Asian-American, and Hispanic-American. Note that White and African-American respondents are of non-Hispanic origin. This leaves us with only 63 respondents that identified as mixed-race out of 3,398 respondents, which provides far too small of an N to estimate our quantities of interests. As a baseline, our smallest racial group (Asian-Americans) is over double that N with 134 respondents. As such, we are left with four racial categories to assess our hypotheses and, as mentioned later in this memo, Pew survey weights to generate covariate estimates of interest that are reflective of the known national population of the United States. We also update the language in the revised manuscript from the term citizens to say "people." We also thank the reviewer's comments regarding the study's scope. We intended this study to be limited to the United States and as such we have updated our title of our study.

> *Is there an indicator of assets the authors could draw on? They use income as an indicator of SES but given historical events, some of these groups have had less of a chance to accumulate wealth and ideally that would be controlled for etc.*

> *More care could have been taken around figure quality and labelling. Please pay a bit more attention to detail and revise.*

> *Is there more info about the sample? Even just a few sentences would help as not all readers are American or know much about this panel.*

We thank the reviewer for these suggestions. First, we do rely on two key indicators of SES as control covariates in our empirical models. Following the traditional approach of controlling for SES, we control for respondent income and education as predictors of COVID-19 social distancing policy support. These are the only two measures of SES within the survey data provided by Pew. We also revise the figures and labelling in the new manuscript revision and provide a few sentences about our nationally representative survey sample used in our study. As to the quality of the figures, for some unknown reason the submission materials include low-resolution images instead of the high resolution images (.tif format) that PLoS One's PACE converter provides. We presume that the final, published versions of the figures will utilize the high-quality .tif format images.

2.3 Implications & Conclusion

I would encourage the authors to expand a bit more re policy implications and potential interventions. What have others suggested here?

We thank the reviewer for this comment and suggestion. We have added in a discussion in our conclusion on possible prescriptions moving forward. In particular, we look at evidence from political science and economics that underscores the importance's of shared-faith and diversity within the scientific community on trust behavior.

3 Reviewer 2

I would like to commend the authors on producing an interesting and important manuscript. I have a number of questions and comments about the manuscript. I hope these are not seen as burdensome or pedantic. My approach to reviewing is to treat the manuscript as though it was given to me by a close colleague who is interested in producing the strongest work possible, and my suggestions are given in that spirit. Especially when there are both multidisciplinary readers and reviewers, some things that may seem "obvious" to the authors may need additional explanation to be parsed by experts from other fields.

3.1 Introduction & Framing

1. P1, L7: Can you clarify the statement about essential workers? The surrounding sentences exclusively address racial and ethnic minorities. It is unclear whether the authors have (or cite) evidence that racial and ethnic minorities are more likely to be classified as essential workers (thus there is a connection), whether this is an assumption, or whether this is just a helpful aside (in which case, perhaps some restructuring would be useful).

We thank the reviewer for noting the confusing language, and in response we have clarified the statement. Specifically, the citation finds that essential workers who are racial/ethnic minorities are more likely to die from COVID-19 than those who are not.

2. P1, LL9-10: Are the data from DC linked to a specific reference? If so, please cite, and if not, please add a reference.

We thank the reviewer for noting this, we have added a clarifying citation.

> *3. P1, LL10-13: Two questions here. First, I think you need to more slowly walk the reader through the logic here. Can you explain in another sentence or two why the higher COVID-19 death rate is consistent with a lack of trust in immunization programs, given that for most of the pandemic vaccinations were not yet available? Second, "trustworthy" implies being worthy of trust, so I assume you mean that they tend to "find" medicine to be less trustworthy?*

We thank the reviewer for noting the confusing jump in logic and misuse of "trustworthy." We have changed the sentence(s) to note that with the ongoing mass vaccination campaign in the US that there is hope of remedying these racial disparities, yet research on Black Americans' distrust of the medical community generally and vaccines specifically may lead to these disparities persisting or even worsening. We also changed "trustworthy" to "trusting."

> *4. P2, LL17-18: Is it accurate to focus exclusively on anti-lockdown protests? What about anti-mask protests and activities (of which there is a surprisingly high prevalence as well)?*

We thank the reviewer for the note to include anti-mask protests and activities, we have added additional language in that vein. Initially, we considered anti-mask protests to be included in anti-lockdown protests, but we agree that including explicit language on those protests and behaviors is warranted.

> *5. P2, LL25-26: I am concerned that this statement may unintentionally be misleading. The cited study indicating increased public trust in science (Sibley et al) was conducted in New Zealand, which is sufficiently different than many other countries' that it may be difficult to generalize even to the US. Several studies sampling from the US have found that trust in science neither increased nor decreased during the pandemic. Another study examining epidemics from 1970 to 2018 found that the general international trend is a decrease in trust in science post epidemic exposure. A Dutch study found something more in line with New Zealand (small increase in trust)*

We thank the reviewer for pointing out other research that has been conducted on public trust in science during the pandemic. We have provided a more balanced perspective and cited research that indicates a positive relationship as well as a negative relationship between scientific trust and the pandemic.

> *6. P2, LL27-29: I am aware of at least 1 study that examined this question, though it is currently a preprint.*

We thank the reviewer for bringing this working paper to our attention. We add the preprint citation to our revised manuscript.

> *7. P2, LL36-37: This was also found in Agley, except that it was linked to liberal/conservative orientation rather than political party (conservatives generally had lower trust in science).*

We thank the reviewer for providing us with an additional and important citation. We include the additional citation in the revised manuscript.

> *8. P2, LL48-49: The use of parentheses in this sentence is a little confusing; the reader may confusedly assume that you are indicating that conservative media is liberal when you write "conservative (liberal) media" - since we often use the formulation "topic or phrase (restatement another way)."*

We have updated the sentence here and broken it down into two sentences to provide more clarity.

> *9. P3, LL74-77: An article from a few years ago [6] argued for a more multifaceted conceptualization of trust in science. Though it may not be necessary to unpack everything in that article (or even to agree with it), I also think there may be more than confidence at play with this variable.*

We thank the reviewer for this interesting article on scientific trust. We have incorporated the article into our introduction to highlight the complex nature of trust.

> *10. P3, LL97-99: Has any study examined whether Tuskeegee is an underpinning factor? I agree that it makes conceptual sense, but one might hesitate to assert that as a core/primary example without evidence (especially in light of other issues related to discrimination)*

We agree with the reviewer that there are other more present-day examples that build off of the Tuskegee study. As such, we include a discussion on the more modern examples of discrimination and medical distrust.

> *11. P4, LL153-155: The use of the word "result" here suggests causality (that is, that higher percentages of Black and Hispanic populations caused higher death counts/rates of daily change). However, it does not seem that the model is designed to permit causal inference.*

We thank the reviewer for this comment and revise the manuscript to reflect the lack of causal inference in our aggregate model assessing the relationship between county-level demographics and COVID-19 mortality.

> *12. P5, Table 1: The finding for Asians in the first daily death count model is markedly different than in models 2 and 3. Do you have any sense of why this might be the case? It is a little unclear whether the only difference between models 1 and 2 is the lagged DV. If so, why would that have such a differential effect?*

We revise the manuscript models in Table 1 to account for the editor's comment regarding missing data, which strengthens the empirical relationship mentioned by the reviewer in that greater proportion of the county-level Asian-American population correlates with less cumulative daily death counts within a county throughout the course of the pandemic.[4] Our county-level models

[4] Note that we also correct our models for multicolinearity, given that the lagged dependent variable was correlated with the dependent variable at $\rho = 0.99$. To that end, and congruent with editor, we add additional language regarding the model specifications in Table 1 and citations to justify our panel modeling approach.

modeling cumulative daily death county and daily death counts are consistent in this finding. We also add additional language clarifying the panel model specification approach and better integrate the model results to our substantive theoretical framework.

> *13. P5, LL161-165: I understand that this is a preliminary analysis designed to set the stage for your main study. At the same time, it is still important to describe limitations and what should - and should not - be intuited based on the findings.*

We thank the reviewer for this important suggestion. In a similar vein as the previous comment regarding the lack of causal inference in our aggregate model analysis, we add additional language describing the limitations of our descriptive finding that higher minority populations are associated with greater COVID-19 mortality at the county-level model.

3.2 Methods

> *14. P6, L178: What is meant by the term "scientific elite""?*

We thank the reviewer for their clarification question, we have changed "elite" to "community."

> *15. P6, LL170-208: I understand that the factor analysis is not your primary analysis, but I am concerned that the reader may not fully understand the decisions that you made based solely on Figure 1 and the current text. For example, reviewing Supplemental Figure 3 without additional text, it is not clear why you selected a single factor rather than a 2-factor solution. Please strongly consider sharing other information about your decision-making (you likely already have all of it in your output files) to improve interpretability. I find Dr. Neill's work out of Australia to be a very clear approach to this.*

We thank the reviewer for their recommendations about clarity surrounding the factor analysis. We added language explaining our decision to use the first dimension from the factor analysis and we have added a factor loadings table and descriptive and reliability statistics table to the supporting information, based on the recommendations/specifications of Neill (2008).

> *16. P7, LL223-224: As with the previous comment, I believe that it would be useful to readers if you shared additional details to improve interpretability of the IRT analysis. For readers in medical sciences, especially, who may not be as familiar with IRT models, this clarification (e.g., even something as simple as the range of composite scores being moved from Table S1 to the main text) will help the reader understand your variable.*

We thank the reviewer for this suggestion. We revise the manuscript to better explain the usage of an IRT model for social distancing policy support, also moving Fig S1 into the main manuscript (now labelled as Fig 2). We add additional reference to Table S1 to direct readers to additional descriptive information about or various dependent variables.

3.3 Results

17. General: While the figures are very helpful visual tools, please consider providing tables with precise outcomes and confidence intervals, as well as precise p values rather than cutoff ranges (e.g., see the American Statistical Association Statement on p-values).

18. P10, LL346-348: There are quite a number of different models being run with both a composite policy score and individual policy scores. One reason for asking for additional prevision (as in my prior comment) is that it is not fully clear how many models were run, which is especially important given the potential for inflated risk of Type I error with multiple comparisons within the same dataset. This is not to say that you need to apply a blanket correction (e.g., Bonferroni), but it is to say that additional information is needed to assist the reader in reviewing and interpreting your findings.

We thank the reviewer for this comment and we add tabular representation of our results as suggested to the supporting information, with references to these tables in the main text, which clarifies the number of models run and highlights further our consistent results. In that vein, we also note in the main text itself that we find consistent support for our main theoretical expectation that scientific trust is associated with increased support for social distancing policies across models with differing outcome variables. This provides relatively robust evidence for our theoretical framework.

3.4 Discussion

19. P10, LL374-376: You indicate that scientific trust "increases support," implying causality, but it is not clear that your study allows for this inference. It may be more accurate to say that latent scientific trust is associated with support.

We thank the reviewer for this distinction in language and we revise the language to reflect the reviewer's suggestion given our lack of causal identification.

20. P10, L376: It may be a bit strong to state that this study itself "confirms" the importance of scientific trust. A more cautious interpretation would be that it provides further robust support for the hypothesis that...

We thank the reviewer for noting the slightly strong language and in response we replace "confirms" with "underscores."

21. P11, LL380: While I agree with your statement that increasing trust in science has the potential to increase support for social distancing policies, there is a substantive gap between support for a policy and compliance with that policy. This study would not seem to provide support for the latter, though it makes sense to suggest it as a future direction for research.

We thank the reviewer for this important question, especially as it was also brought up by the editor. In the introduction of our manuscript we add a discussion on the relationship between compliance

and support for government policies. We also ground this discussion in the representational literature, arguing that government action requires support from the mass public. For more information on this comment please refer to our general comment responses to the Editor.

> *22. P11, LL383-385: Can you clarify what you mean by a "Black-specific intervention"? This study examines support for broad societal-level policies, but this language here implies that increasing trust in science among a specific racial group would actively reduce infection rates and mortality. That is a fairly substantial leap that I am not sure can be justified.*

We thank the reviewer for this comment. We have changed the framing of this sentence so that it is now focused on the policy support. By this we mean that an intervention (e.g. to increase scientific trust) among the African American community may increase support policies recommended by scientists.

> *23. P11, LL391-400: Another limitation, as you yourselves point out, is that you measured trust in early 2019, and policy support in March 2020. While some research suggests that trust in science may not have changed during that time, especially in the US, international data suggest different trends.*

We thank the reviewer for this suggestion and context. The reviewer is correct, there is mixed evidence that aggregate trends in scientific trust differ prior and after the onset of the COVID-19 pandemic. Within the United States, panel data shows that the mass public's trust in science did not shift from December 2019 to March 2020 (Agley 2020).[5] This finding within the United States is replicated in another panel dataset (Luna et al. 2020).[6] Moreover, the reviewer is correct that this finding of stability in scientific trust is mixed in the comparative case (Eichengreen et al. 2021; Oude Groeniger et al. 2021).[7] [8] We contend that within the polarized context of the United States, that a pre-pandemic measure of latent scientific trust is a source of strength given that this measure is *endogenous* to support for social distancing policies aimed at slowing the spread of the COVID-19 pandemic. Nevertheless, we concur with the reviewer that this may be a limitation and we added some language citing the research highlighted by the reviewer that scientific trust in the American public is most likely stable throughout the pandemic.

4 Editor's Review Comments

> *First, let me be clear: I believe that your manuscript is of high quality and it was a real pleasure to read it. As you will see, the reviewers are also quite positive, but outline*

[5] Agley, J. (2020). Assessing changes in US public trust in science amid the COVID-19 pandemic. Public Health, 183, 122–125.

[6] Luna, D. S., Bering, J. M., & Halberstadt, J. B. (2021). Public faith in science in the United States through the early months of the COVID-19 pandemic. Public Health in Practice, 2(December 2020), 100103.

[7] Oude Groeniger, J., Noordzij, K., van der Waal, J., & de Koster, W. (2021). Dutch COVID-19 lockdown measures increased trust in government and trust in science: A difference-in-differences analysis. Social Science and Medicine, 275(March), 113819.

[8] Eichengreen, B., Aksoy, C. G., & Saka, O. (2021). Revenge of the experts: Will COVID-19 renew or diminish public trust in science? Journal of Public Economics, 193, 104343.

many comments. R1 has many very useful suggestions and comments that, if taken into account, would improve the manuscript. I should, however, mention that you can use the word 'result' although, following the spirit of R1's comment, you should be clear that you do not uncover a causal relationship. R2 also provides several useful comments. Among others, I agree that clarifications are needed regarding the different measures/scales used. On top of R2's points, I should add that the items for 'policy support' should reflect the exact wording. I assume that it is currently not the case, notably because of the wording of ">10 people" (which would probably not be written that way in the survey). Still on policy support, you mention that the outcome is dichotomized, but was that the initial scale (i.e. necessary versus unnecessary) or did you simplify the original scale? Overall, I invite you to address both reviewers' comments and I believe that they will allow you to resubmit a stronger manuscript. Below, I also provide my own comments:

4.1 General Comments

Is policy support the most important outcome to measure or would not it be more useful to consider levels of compliance with public health preventive measures? I assume that compliance it more closely linked to the disproportionate number of infections and deaths among different subgroups of the population. You briefly acknowledge this in the discussion (e.g. "Specifically, increasing trust in science within communities of color has the potential to significantly increase support for and potentially compliance with social distancing policies specifically"), but it would benefit from more details. Put it simply: do you rely on the assumption for policy support is a very strong predictor of compliance?

We thank the editor for their kind words regarding our manuscript and for this important point. Indeed, our theoretical framework posits that mobilizing mass public support, particularly across racial groups, for public health policies that limit the spread of COVID-19 infection is critical in the ongoing societal effort to halt the pandemic. This is of heightened importance given the standing inequality that exists in COVID-19 mortality rates within the United States, with communities of color bearing the disproportionate number of COVID-19 attributed deaths throughout the course of the pandemic. In that vein, we argue that scientific trust—as opposed to institutional or media trust—can provide a critical mechanism towards increasing support for policies aimed at stopping the spread of COVID-19 and, ultimately, the racial inequities exposed by the pandemic.

We agree with the reviewer that our theoretical framework requires more discussion towards distinguishing between policy support and compliance. While we agree that compliance is an important outcome variable, we argue that in order to overcome the stark collective action problem posed by stopping transmission of COVID-19, it is critical that government pass policies congruent towards that end. Indeed, recent work by Wibbens, Koo, & McGahan (2020) show that implementation of COVID-19 policies aimed at ensuring social distancing in society were critical in reducing overall COVID-19 transmission rates in 40 countries and across U.S. states.[9] This work explicitly argues that, in the absence of government intervention and reliance only social distancing practices, rates of COVID-19 infections and deaths would have been much higher. As such, we situate our theoretical framework in standard representational terms, that public

[9]Wibbens, P. D., Koo, W. W. Y., & McGahan, A. M. (2020). Which COVID policies are most effective? A Bayesian analysis of COVID-19 by jurisdiction. PLoS ONE, 15(12 December), 1–19.

support for policies is a critical condition for government implementation of policies that overcome societal collective action problems such as stopping the transmission of COVID-19. We add more of this context to our revised manuscript in both the motivating introduction and discussion. In our revision, we also note the high correlation between compliance between social distancing policies and support for government policies ensuring such policies. For example, Schmelz (2020) finds stark evidence that social compliance of social distancing practices is highly correlated with support for government action aimed at ensuring compliance with social distancing within the context of Germany.[10] This finding of a high correlation between compliance and policy support is replicated in other studies within the United States (Czeisler et al. 2020) and the Netherlands (Kuiper et al. 2020).[11] [12]

Taken together, we thank the editor for this important point strengthening our theoretical framework by situating it more clearly within a representational context by noting that increased support for policies are a critical component towards facilitating passage of government policies aimed at overcoming collective action problems. We also note in the revision that while the correlation between compliance with social distancing and support for policies ensuring social distancing, that ultimate mitigation of the collective action problem posed by the spread of the COVID-19 pandemic relies on government policy interventions. We also revise the manuscript to reflect the original question wording and scales used to measure policy support, which was measured in the initial "support" or "oppose" scales across policies.

> *As for citizens' compliance with preventive measures, social desirability bias that might at play. The literature is mixed. For example, research using list experiments like Larsen et al. (2020) suggests that it is not an issue, while other research who used a face-saving (or 'guilt-free') find evidence or such a bias. Your manuscript would benefit from addressing this potential issue, and most importantly whether it would only affect the distributions of your outcomes (on policy support) or also your inferences.*

We thank the editor for highlighting this potential problem and in response have added significant discussion of social desirability bias in the "Social distancing policy support" subsection. Based on the evidence discussed there, we believe that it is highly unlikely that our results are biased by social desirability in such a way as to complicate our substantive results.

> *You include median age in your model, as well as the percentage of 65+ years old. It is not clear why you need both, and I was wondering if there was a rationale behind it that was currently missing.*

We believe that including median age and percentage of 65+ years old is necessary as they are distinct measurements. Specifically, 65+ measures how critically vulnerable a given county is, as all COVID related research suggests that age is one of the most influential factors in the mortality rate of a COVID infection, whereas the median population measures the general vulnerability of

[10] Schmelz, K. (2020). Enforcement may crowd out voluntary support for COVID-19 policies, especially where trust in government is weak and in a liberal society. Proceedings of the National Academy of Sciences of the United States of America, 118(1).

[11] Czeisler, M. É., Howard, M. E., Robbins, R., Barger, L. K., Facer-Childs, E. R., Rajaratnam, S. M. W., & Czeisler, C. A. (2020). COVID-19: Public Compliance with and Public Support for Stay-at-Home Mitigation Strategies. MedRxiv.

[12] Van Rooij, B., De Bruijn, A. L., Folmer, C. R., Kuiper, M. E., Brownlee, M., Olthuis, E., & Fine, A. (2020). Compliance with COVID-19 Mitigation Measures in the United States. PsyArXiv, 1–40.

the county, the higher the number the more overall threat. Age is most important above 65, but a county full of 20 year olds is still significantly less at risk than a county full of 55 year olds, which the 65+ measure would fail to capture but the median age measure would better explain. Finally, our results do not substantively change when only one or both of the measures are used in our analysis.

> *Clarification: why is the N for the observation level varying across the different DVs (Table 1)?*

We thank the editor for this clarification detail regarding the N observation level varying across DVs. We have corrected this minor error in our revised manuscript and now all models feature a full set of observations in our dataset observing 3,142 counties over 346 days of the pandemic from the first reported case on January 21st in Snohomish County, WA until December 31st (346 days × 3,142 counties = 1,087,132 observations). Our model results remain unchanged from the previous iteration of the manuscript.

> *Given the size of the coefficients, Models 3-6 from Table 1 should have more than two decimals (or you could change the scale of the IV, but you might prefer to simply show more decimals). For example, the effect for White is always 0.00 (0.00) but is said to be statistically significant in model 6. Related to comment #17 of R1, it provides a misleading understanding of statistical significance.*

We concur with the editor that simply changing the decimal points would better convey the results of our daily change models (models 3–6) and our revised manuscript now reflects this change from two to three decimal places. We also clarify our methodological approaches in modeling the relationship between county-level demographics/ethnicity and the severity of COVID-19 death rates in the manuscript, which also helps address some of R1's points regarding greater methodological clarity behind the county-level models. Specifically, we spend more time walking the reader through our county-level models and how our models help assess cross-sectional county differences in cumulative and daily change COVID-19 death rates on the basis of county-level demographic variation. We also elaborate that these models leveraging the entire 2020 pandemic year help assess empirically—in a comprehensive fashion that is often lacking in standing studies of COVID-19 disparities—that racial disparities exist with respect to COVID-19 death rates. Simply put, we use these models showing that counties with greater minority communities were associated with greater COVID-19 deaths to motivate the significance of our individual-level models finding that increasing scientific trust across all racial groups is essential to garnering support for public health policies that lessen the severity of the current, and potentially a future, pandemics that disproportionately affect communities of color.

> *The following work would likely benefit your discussion on the notion of trust: Devine, D., Gaskell, J., Jennings, W., & Stoker, G. (2020). Trust and the Coronavirus Pandemic: What are the Consequences of and for Trust? An Early Review of the Literature. Political Studies Review, 1478929920948684.*

We thank the editor for this important citation and we have incorporated it into the introduction of our paper.

Clarification on line 196: "also differences in this measure across racial groups." Sounds like the factor loading is different based on subsamples, but then we understand that you simply compare the means of this measure across these subsamples. It could be clearer right away.

Clarification needed, line 243: To explain the "appropriate survey weights." The Figures should include a note explaining what the CI reflect. I see that there are 2 levels. Are they, for example, 90 and 95% CI? To clarify.

We thank the reviewers for these comments for elaboration on these important points in the manuscript. We clarify language in the manuscript articulating that we are comparing mean values of latent scientific trust across racial groups rather than comparing factor loadings across groups. We also revise the manuscript to explain the appropriate survey weights used in our empirical models on line 243. Specifically, we add language regarding the Pew survey weights used in our study and how these survey weights are used to correct for non-response bias by providing for a sample that is representative of the known national population on such characteristics such as age, gender, education, race, and foreign-born nationality. Indeed, we apply these survey weights derived by Pew to generate our model estimates that help alleviate concerns regarding whether these estimates are representative to the general population. We also concur with the point regarding the clarification on the levels the confidence intervals articulate; the editor is indeed correct that the intervals reflect 90% and 95% confidence levels relevant for statistical inference hypothesis testing, respectively. We follow the editor's suggestion by adding a note in each figure explaining that thick point-estimate bands mark 90% confidence levels and thin point-estimate bands make 95% confidence levels.

To reiterate, we greatly appreciate the feedback provided by both reviewers and the editor. We believe that the edits that we've made in response have greatly increased the quality of our manuscript. We look forward to hearing your responses to the revised manuscript and this memo.
